# Utilisation of ChatGPT and other Artificial Intelligence tools among medical faculty in Uganda: a cross-sectional study

**DOI:** 10.12688/mep.20554.3

**Published:** 2025-04-28

**Authors:** David Mukunya, Ritah Nantale, Frank Kayemba, Elizabeth Ajalo, Kennedy Pangholi, Jonathan Babuya, Suzan Langoya Akuu, Amelia Margaret Namiiro, Ronald Tweheyo, Steven Ekak, Brenda Nakitto, Kirsten Nantongo, Joseph Luwaga Mpagi, Milton W. Musaba, Faith Oguttu, Job Kuteesa, Aloysius Gonzaga Mubuuke, Ian Guyton Munabi, Sarah Kiguli

**Affiliations:** 1 Department of Community and Public Health, Busitema University-Mbale Campus, Tororo, Eastern Region, Uganda; 2 Medicine, Mbarara University of Science and Technology Faculty of Medicine, Mbarara, Western Region, Uganda; 3 Department of Pediatrics and Child Health, Makerere University School of Health Sciences, Kampala, Central Region, Uganda; 4 Faculty of Health Sciences, Gulu University Faculty of Science, Gulu, Northern Region, Uganda; 5 Department of Microbiology and Immunology, Busitema University-Mbale Campus, Mbale, Uganda; 6 Obstetrics and Gynaecology, Busitema University -Mbale Campus, Mbale, Uganda; 7 Department of Surgery, Mulago National Referral Hospital, Kampala, Central Region, Uganda; 8 Department of Radiology, Makerere University School of Health Sciences, Kampala, Central Region, Uganda; 9 Department of Human Anatomy, Makerere University School of Health Sciences, Kampala, Central Region, Uganda

**Keywords:** ChatGPT, medical faculty, Bing, Bard, Uganda, generative AI, medical education

## Abstract

**Background:**

ChatGPT is a large language model that uses deep learning techniques to generate human-like texts. ChatGPT has the potential to revolutionize medical education as it acts as an interactive virtual tutor and personalized learning assistant. We assessed the use of ChatGPT and other Artificial Intelligence (AI) tools among medical faculty in Uganda.

**Methods:**

We conducted a descriptive cross-sectional study among medical faculty at four public universities in Uganda from November to December 2023. Participants were recruited consecutively. We used a semi-structured questionnaire to collect data on participants’ socio-demographics and the use of AI tools such as ChatGPT. Our outcome variable was the use of ChatGPT and other AI tools. Data were analyzed in Stata version 17.0.

**Results:**

We recruited 224 medical faculty, majority [75% (167/224)] were male. The median age (interquartile range) was 41 years (34–50). Almost all medical faculty [90% (202/224)] had ever heard of AI tools such as ChatGPT. Over 63% (120/224) of faculty had ever used AI tools. The most commonly used AI tools were ChatGPT (56.3%) and Quill Bot (7.1%). Fifty-six faculty use AI tools for research writing, 37 for summarizing information, 28 for proofreading work, and 28 for setting exams or assignments. Forty faculty use AI tools for non-academic purposes like recreation and learning new skills. Faculty older than 50 years were 40% less likely to use AI tools compared to those aged 24 to 35 years (Adjusted Prevalence Ratio (aPR):0.60; 95% Confidence Interval (CI): [0.45, 0.80]).

**Conclusion:**

The use of ChatGPT and other AI tools was high among medical faculty in Uganda. Older faculty (>50 years) were less likely to use AI tools compared to younger faculty. Training on AI use in education, formal policies, and guidelines are needed to adequately prepare medical faculty for the integration of AI in medical education.

## Introduction

ChatGPT is a large language model that uses deep learning techniques to generate human-like texts to user prompts ^1^. Over 500 million people all over the world actively use ChatGPT in various fields including medical education ^2^. ChatGPT and other Artificial Intelligence tools such as; - Bard/Gemini, Bing, Desktop, Write Sonic, Klap, Jasper, and Quilt Bot AI have the potential to revolutionize medical education as they act as interactive virtual tutors and personalized learning assistants ^3– 5^. In developing countries, faculty face several challenges such as limited time to manage multiple tasks like lesson planning, and grading ^6^. There are also few faculty as compared to the available students ^6^. These challenges can potentially lead to stress and burnout, which can ultimately affect the quality of teaching ^7^. In addition, institutions face the challenge of limited resources such as textbooks, technology, and classroom materials, to provide students with the best possible learning experience ^6^. ChatGPT and other AI tools can be powerful tools to help educators and institutions overcome these challenges. These AI tools can generate teaching content (questions and answers, quizzes, and assignments) for educators and assist them in grading students ^8^. This can save time and effort of educators to focus on other areas of teaching ^8^. Despite the great potential of AI tools such as ChatGPT, there are various concerns regarding their use in medical education including plagiarism, incorrect or fabricated responses, copyright and privacy infringements and security concerns ^9– 12^. Thus, educators need to learn not only how to use AI tools but also how to use them responsibly. There is a need to establish whether educators are using AI tools. This is will inform policymakers to develop guidelines to promote responsible use of AI tools in medical education. However, there is limited information about the use of ChatGPT among medical faculty in low- and middle-income countries. We conducted our study to find out the prevalence of use of artificial intelligence tools among medical faculty, the different AI tools used by medical faculty, and factors associated with the use of AI tools among medical faculty in four public universities in Uganda.

## Methods

### Study design

This was a descriptive cross-sectional study aimed at assessing the use of ChatGPT and other Artificial Intelligence (AI) tools among medical faculty in Uganda.

### Study setting

The study was conducted at four public universities in Uganda. These included Busitema University, Makerere University, Mbarara University of Science and Technology, and Gulu University. These Universities were selected because they are the largest and oldest public universities that offer medical degrees in Uganda.

Busitema University Medical School was established in 2013 and is located in Mbale city, eastern Uganda. Theschool has 54 faculty, 13 departments, and provides medical education to about 550 students at undergraduate and postgraduate levels.

Makerere University Medical School was founded in 1924 and is located on Mulago Hill in north-east Kampala, Uganda's capital and largest city. The school has 86 faculty, 14 departments, and provides medical education to about 1310 students at undergraduate, and postgraduate levels.

Mbarara University of Science and Technology was founded in 1989 and is located on the premises of Mbarara Regional Referral Hospital, in the city of Mbarara, Western Uganda. The school of medicine has 100 faculty, 25 departments and provides medical education to about 1400 students at undergraduate, and postgraduate students.

Gulu University Medical School was founded in 2004 and is located Gulu city, the largest urban center in Northern Uganda, approximately 345 kilometers (214 mi), by road, north of Kampala, Uganda's capital and largest city. The school has 64 faculty, 13 departments, and provides medical education to 490 students at undergraduate, and postgraduate levels.

### Study population

Medical faculty involved in teaching students both clinical and non-clinical disciplines. These included teaching assistants, lecturers, senior lecturers, professors, and deans of faculty.

### Eligibility criteria

We included medical faculty at the selected universities who consented to participate in the study between November 2023 and December 2023. Medical faculty not available during the data collection period were excluded.

### Study variables

Our outcome variable was use of AI tools such as ChatGPT among medical faculty. Participants were asked if they have ever used AI tools such as ChatGPT, this was recorded as Yes (denoted as 1) or No (denoted as 0). The independent variables included; socio-demographic characteristics (age, sex, religion, marital status, university, department, work experience) and being aware of AI tools.

### Data collection tool and procedures

A semi-structured questionnaire was developed including participant demographics, and a list of AI tools. The AI tool list in this study was based on a qualitative study– unpublished – exploring the AI tools used by medical faculty and students in Uganda, all AI tools mentioned in that study were included in the questionnaire.

Data were collected by eight trained research assistants using a semi-structured standardized questionnaire. During the study period, research assistants scheduled in-person appointments with individual faculty. Informed written consent was obtained, a hard copy questionnaire was provided by the research assistant and filled by the faculty at a convenient time within 3 weeks. The faculty contacted the research assistant to pick up the answered questionnaires.Data entry was done by research assistants using an electronic data collection form designed in Kobo Toolbox (Cambridge, Massachusetts, USA). Kobo Toolbox is an open-source software developed by the Harvard Humanitarian Initiative with support from United Nations Agencies, CISCO, and partners to support data management by researchers and humanitarian organizations ( https://www.kobotoolbox.org/).

### Sample size estimation and sampling technique

We aimed to recruit at least 80% (243) of the 304 faculty at the 4 colleges of health sciences of the selected universities. We were able to reach 224 faculty, (19) were unreachable during the time of the study. Due to the limited study population, we used consecutive sampling.

### Data analysis

Data were cleaned and analyzed in Stata version 17.0. We summarized categorical data using frequencies and percentages, and numerical data as mean (standard deviation) and median (interquartile range) as appropriate. Bar graphs and pie charts were used for data visualization. We conducted a generalized linear regression for the Poisson family with a log link to estimate prevalence ratios between use of AI tools and various exposures. We used robust cluster variance estimation to adjust for clustering at the University level.

### Ethical consideration

Ethics approval to conduct the study was granted by the Busitema University Research Ethics Committee (approval number: BUFHS-2023-79) on 23/09/2023. Written informed consent was obtained from the participants before recruitment into the study.

## Results

### Participant characteristics

We recruited 224 faculty with majority [75% (167/224)] being male. The median age (interquartile range) was 41(34–50). More than a third 38% (84/224) were Catholics and the least (4%) were Seventh-day Adventists. Regarding departments Internal medicine; and microbiology and immunology had the highest (13% and 12% respectively) number of participants while radiology and nursing had the lowest (2% and 1% respectively). Majority [82% (183/224)] of the participants were married. Almost all [90% (202/224)] had ever heard of AI tools such as ChatGPT. Most [44.1% (89/202)] got to know AI tools such as ChatGPT from social media, 42.1% (85/202) from friends, 15.8% (32/224) fellow lecturer, and 23.3% (47/202) cited other sources including students, and family. Details of participant characteristics are in Table 1.

**Table 1.** Characteristics of medical faculty in Uganda who participated in a survey assessing use of AI tools.

Characteristic	Ever used AI tools such as ChatGPT		
	No (n=82)	Yes (n=142)	Total (n=224)
	n (%)	n (%)	n (%)
**Age**			
24–35	19 (23)	54 (38)	73 (33)
36–50	37 (45)	62 (44)	99 (44)
>51	26 (32)	26 (18)	52 (23)
**Sex**			
Female	19 (23)	38 (27)	57 (25)
Male	63 (77)	104 (73)	167 (75)
**Religion**			
Anglican/Protestant	21 (26)	39 (28)	60 (27)
Pentecostal/Born again	18 (22)	35 (25)	53 (24)
Catholic	35 (43)	49 (35)	84 (38)
Muslim	6 (7)	12 (9)	18 (8)
Seventh-Day Adventist	2 (2)	7 (5)	9 (4)
**University**			
Gulu University	21 (26)	40 (28)	61 (27)
Busitema University	16 (20)	36 (25)	52 (23)
Makerere University	23 (28)	27 (19)	50 (22)
Mbarara University of Science and Technology	22 (27)	39 (28)	61 (27)
**Department**			
Community and Public Health	2 (2)	8 (6)	10 (4)
Biochemistry	8 (10)	9 (6)	17(8)
Obstetrics and Gynecology	7 (9)	7 (5)	14(6)
Nursing	1(1)	2 (1)	3(1)
Internal Medicine	11(13)	19(13)	30 (13)
Microbiology and Immunology	7(9)	19 (13)	26(12)
Anatomy	6(7)	10 (7)	16(7)
Physiology	6(7)	7(5)	13(6)
Pathology	5(6)	15(11)	20(9)
Surgery	12(15)	14(10)	26(12)
Pharmacology and therapeutics	2(2)	10(7)	12(5)
Pediatrics and child health	5(6)	12(8)	17(8)
Family medicine	4(5)	2(2)	6(3)
Radiology	2 (2)	2 (1)	4(2)
Others	4 (5)	6 (4)	10 (4)
**Marital status**			
Single	8 (10)	33 (23)	41 (18)
Married	74 (90)	109 (77)	183 (82)
**Ever heard of AI tools like ChatGPT**			
Yes	60 (73)	142 (100)	202 (90)
No	22 (27)	0 (0)	22 (10)
**Ever recommended AI tool to student(s)**			
Yes	71 (86.6)	58 (40.9)	129 (57.6)
No	11 (13.4)	84 (59.1)	95 (42.4)

### Use of ChatGPT and other AI tools among medical faculty

**Figure 1.**
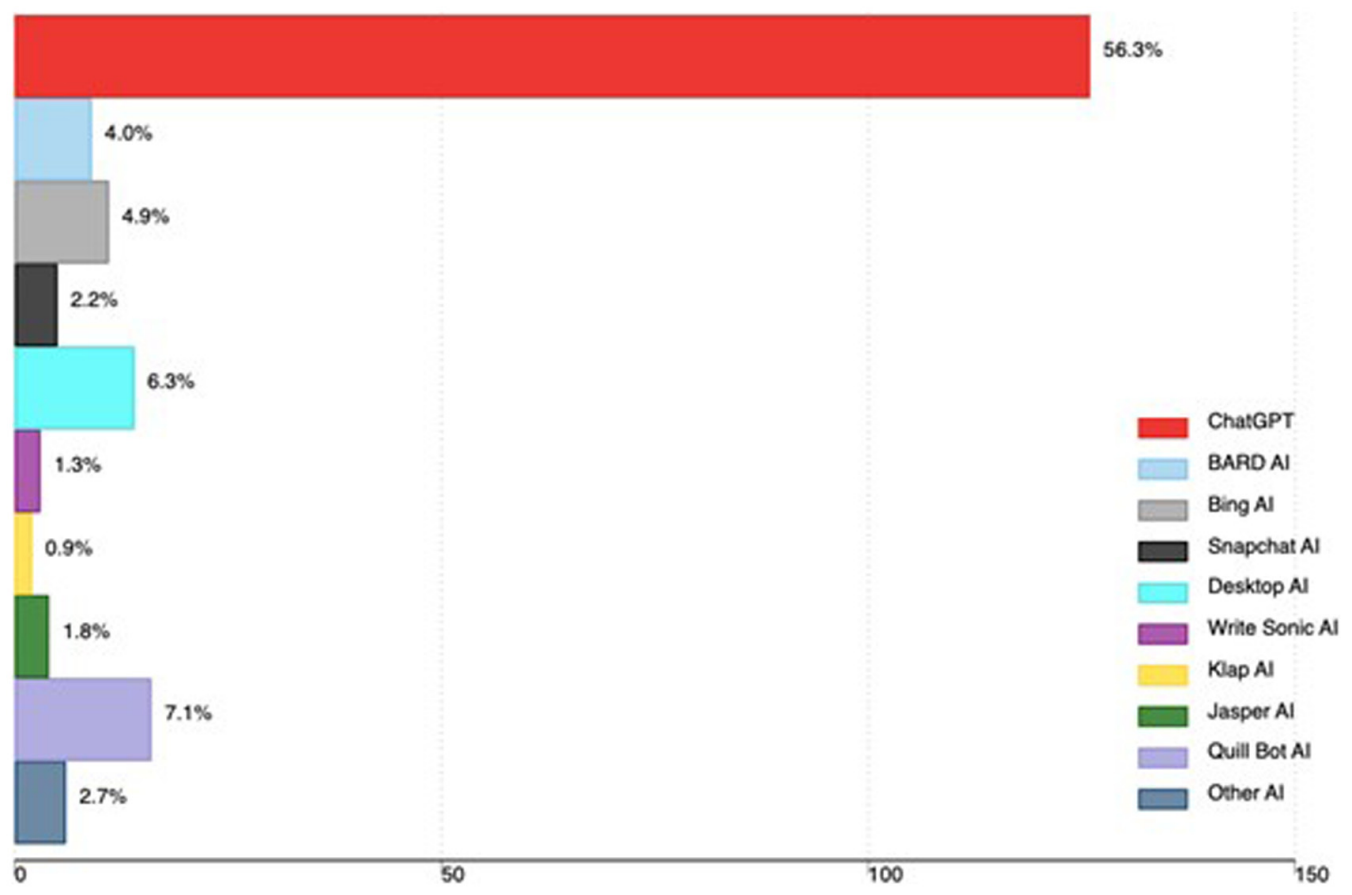
AI tools used by medical faculty in Uganda (the x-axis shows the total number of faculty using AI, and the percentage is based on all participants in the study).

A total of 142 out of 224 participants (63.4%) had ever used AI tools. The majority 56.3% had ever used ChatGPT, 7.1% Quill Bot, 6.3% Desktop AI and 4.9% Bing AI. Other AI tools used by medical faculty are in Figure 1.

**Figure 2.**
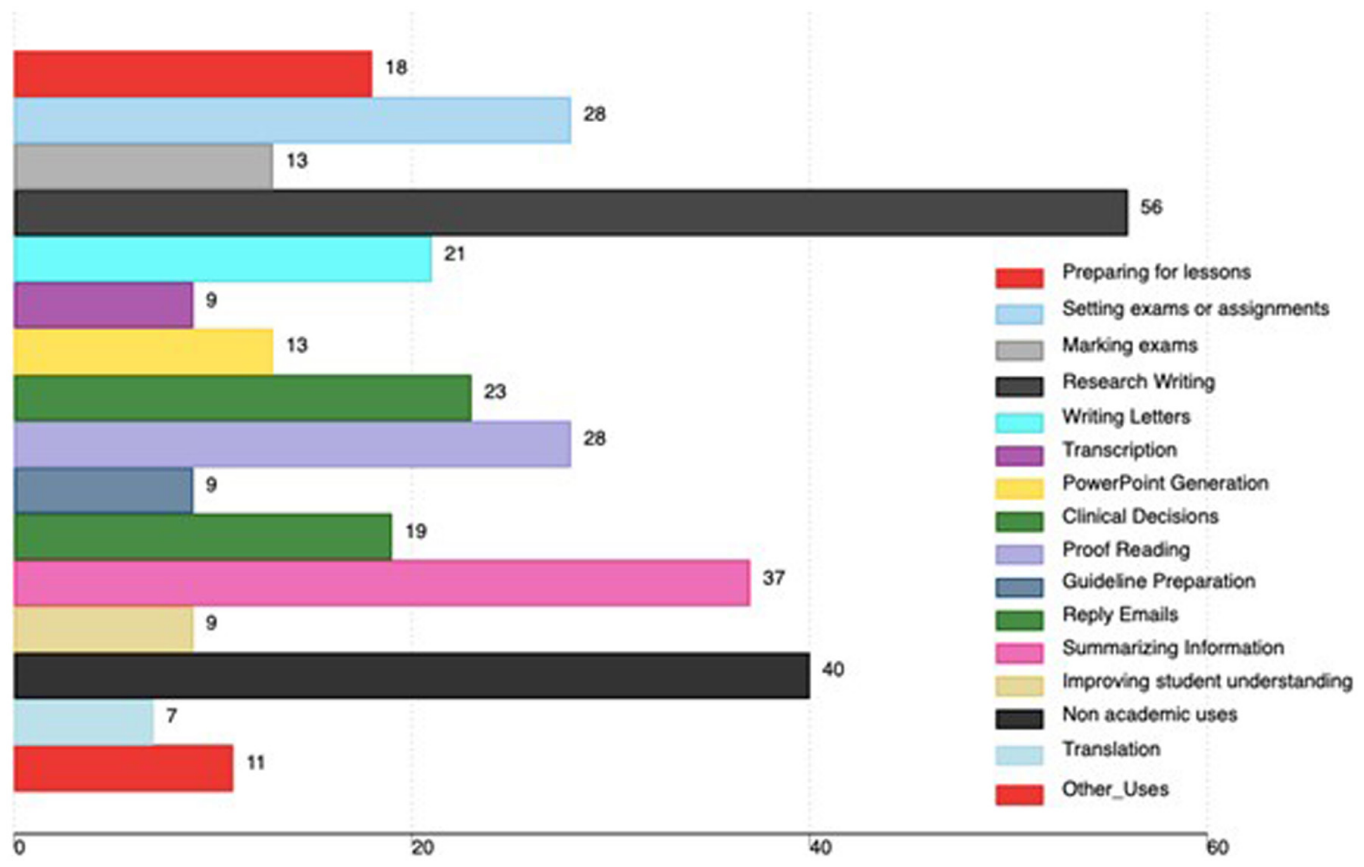
Academic uses of AI tools among medical faculty in Uganda (the x-axis shows the total number of faculty using AI, and the percentage is based on all participants in the study).

Regarding AI tools use, 56 faculty used AI tools for research writing, 37 used AI tools for summarizing information, 28 used AI tools for proofreading work and 28 used them for setting exams or assignments. 40 faculty used AI tools for non-academic uses. Other uses of AI tools are in Figure 2.

**Figure 3.**
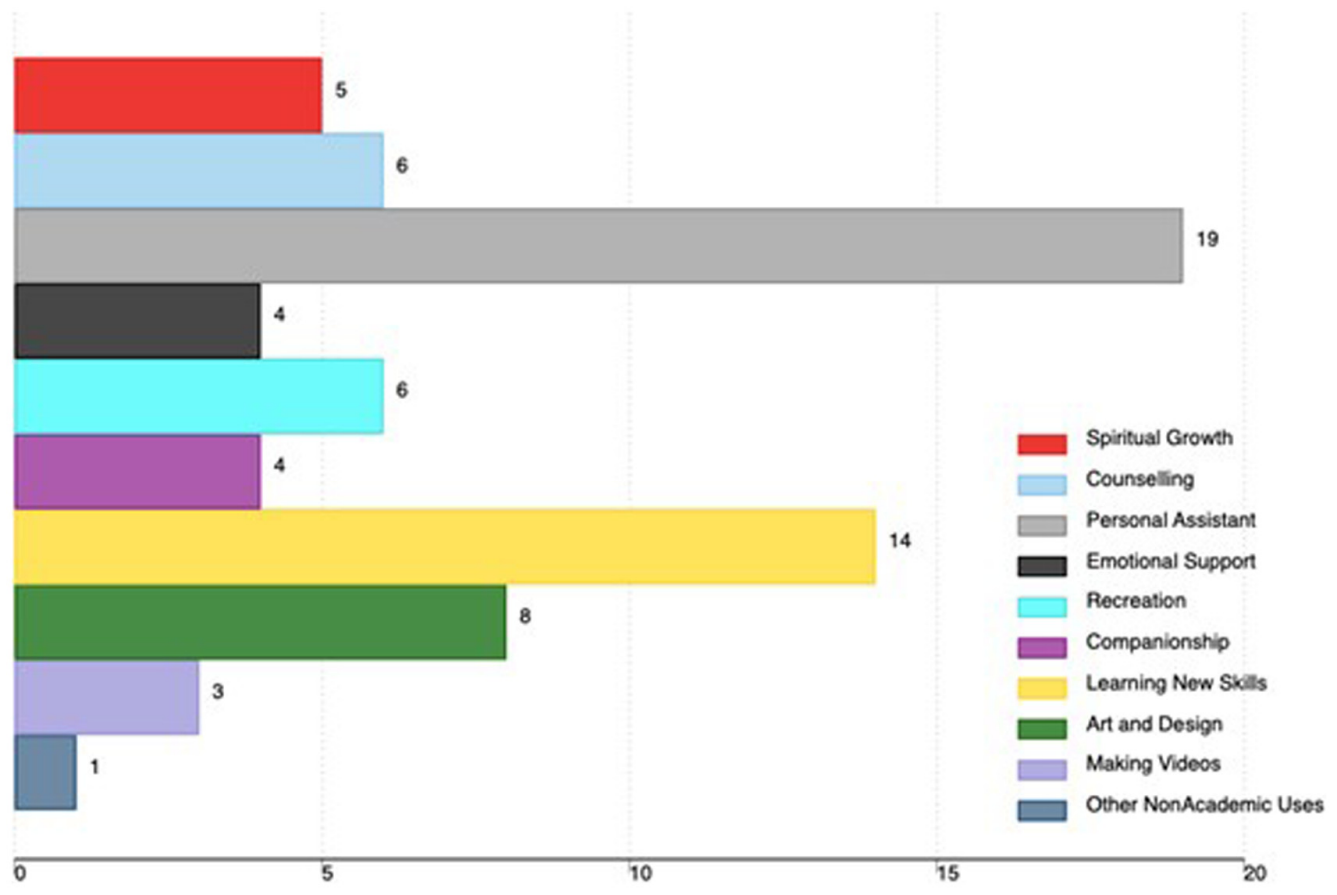
Non-academic uses of AI tools among medical faculty in Uganda (the x-axis shows the total number of faculty using AI, and the percentage is based on all participants in the study).

Non-academic uses included the use of AI tools as a personal assistant (19), learning new skills (14), art and design (8), recreation (6), and counseling (6). Other non-academic uses are in Figure 3.

### Factors associated with the use of ChatGPT and other AI tools among medical faculty in Uganda

Medical faculty aged greater than 50 years were 40% times less likely to use AI tools such as ChatGPT as compared to those aged 24 to 35 years (aPR:0.60; 95% CI: [0.45, 0.80]).

Medical faculty in Makerere University were 32% times less likely to use AI tools as compared to those in Gulu University (aPR: 0.68 95% CI: [0.60, 0.78]). Details are in Table 2.

**Table 2.** Factors associated with the use of ChatGPT and other AI tools among medical faculty in Uganda.

Variable	cPR [95% CI]	P-value	aPR [95% CI]	P-value
**Age in years**				
24–35	1		1	
36–50	0.85 [0.59, 1.22]	0.371	0.80 [0.55, 1.15]	0.23
>51	0.68 [0.58, 0.79]	<0.001	**0.60 [0.45, 0.80]**	**0.001**
**Gender**				
Female	1		1	
Male	0.93 [0.67, 1.31]	0.693	0.92 [0.67, 1.27]	0.619
**Religion**				
Anglican	1		1	
Pentecostal/Born again	1.02 [0.70, 1.48]	0.935	1.01 [0.72, 1.40]	0.976
Catholic	0.90 [0.66, 1.21]	0.484	0.89 [0.70, 1.14]	0.374
Muslim	1.03 [0.82, 1.29]	0.827	1.01 [0.80, 1.27]	0.937
Seventh Day Adventist	1.20 [0.94, 1.53]	0.154	1.18 [0.94, 1.48]	0.145
**University**				
Gulu University	1		1	
Busitema University	1.06 [1.06, 1.06]	<0.001	0.86 [0.72, 1.01]	0.066
Makerere University	0.82 [0.82, 0.82]	<0.001	**0.68 [0.60, 0.78]**	**<0.001**
Mbarara University of Science and Technology	0.98 [0.97, 0.98]	<0.001	**0.80 [0.69, 0.92]**	**0.003**

## Discussion

In this study, we found that the use of AI tools among medical faculty was high (63.4%). The high use of AI tools among medical faculty in our study could be attributed to the increased awareness about AI tools among medical faculty. Almost all faculty in our study (90%) had ever heard about AI tools such as ChatGPT. Contrary to our findings, a study among medical faculty in Nigeria showed that only 32% of the faculty had ever used ChatGPT ^3^. This could be attributed to the fact that the study in Nigeria was conducted in May 2023 ^3^ yet our study was done in November 2023, AI tool visits showed an increasing rate per month in 2023 ^13^.

A study among medical students in Uganda showed a higher (75.7%) AI use among students compared to 63.4% among faculty ^14^. This is consistent the study in Nigeria ^15^. However, a study in Pakistan showed no differences in use of AI among faculty and students, this is possibly due to younger faculty in Pakistan compared to older faculty in Uganda ^16^. Training faculty on using AI increases their interest and may increase use ^15^.

Our study showed a very low user rate of other AI tools as compared to ChatGPT. Similarly, a study in Nigeria showed that use of other AI tools such as Bing, Quill bot and Bard was very low compared to ChatGPT ^3^. This could imply low awareness about other AI tools among medical faculty. Our findings are consistent with a survey of AI tools that showed that ChatGPT took over 60% of the total traffic of the 50 most visited AI tools in 2023 ^13^.

The most prevalent use of ChatGPT among medical faculty was research writing. A study in USA showed text generation and the most common use of generative AI among medical educators though this study didn’t specify use the AI generated text ^17^. Another study among doctoral students in China showed a high intention to use ChatGPT in writing ^18^. Similarly, a study in Saudi Arabia also showed that healthcare workers mostly used AI tools for medical research (69.5%) ^19^. This highlights the great potential of AI tools in academic writing. The high use of AI in research writing could be attributed to the fact that most faculty find research writing very difficult. However, in December 2023, the European Research Council issued a warning against use of generative chatbots in research writing and emphasized that researchers have full responsibility for any plagiarism or fabrication by the chatbots ^20^.

Medical faculty in our study also reported the use of AI tools for summarizing information, proofreading work, and setting exams or assignments. Similar uses have been reported in studies conducted elsewhere ^3, 8, 19^.

Notably, faculty use chatbots for non-academic purposes such as recreation and learning new skills. This is a neglected discussion overshadowed by the academic implications. More studies may be required to understand the impact of AI tools on mental and social life.

Medical faculty aged greater than 50 years were 40%less likely to use AI tools such as ChatGPT as compared to the younger faculty. Similar results have been showed in previous studies ^3^. Younger people are more exposed to AI through social media and other digital platforms thus could be more likely to use AI tools as compared to those older ^3, 19^. Further, older people are often pessimistic and slow to adopt new technology ^21– 23^.

Bigger medical schools like Makerere and Mbarara were less likely to use AI compared to smaller medical schools like Gulu. Generative AI is universally available and no university in Uganda has publicly prohibited or discouraged use of AI, these differences remain unexplained.

### Strengths and limitations

Findings from this study can inform stakeholders about extent of use of AI in medical education by faculty. To the best of our knowledge, this is one of the few studies that have been done to assess use of AI tools such as ChatGPT among medical faculty in low-resource settings. Additionally, we included four universities, one from each region of the country thus our findings can be generalizable to all medical students in the country. Our study also had limitations; sampling was uneven across departments and we didn’t assess for prior training on use of AI tools. Other large language models like Llama, Claude and command R were not included in the questionnaire.

## Conclusion

The use of ChatGPT and other AI tools was high among medical faculty in Uganda. Older faculty (>50 years) were less likely to use AI tools compared to younger faculty. Training on AI use in education, formal policies, and guidelines are needed to adequately prepare medical faculty for integration of AI in medical education.

## Declarations

### Ethics and consent

Ethics approval to conduct the study was granted by the Busitema University Research Ethics committee (Committee (approval number: BUFHS-2023-79).) on 23/09/2023. Written informed consent was obtained from the participants before recruitment into the study. All the study procedures were performed in accordance with the Declaration of Helsinki.

## Consent for publication

Not applicable

## Data Availability

Figshare: Gen AI data. https://doi.org/10.6084/m9.figshare.26489884.v2 ^24^. The project contains the following underlying data: AI data set.xls. Figshare: Gen AI data. https://doi.org/10.6084/m9.figshare.26489884.v2 ^24^. This project contains the following extended data: QUESTIONNAIRE FACULTY.pdf *Data are available under the terms of the Creative Commons Attribution 4.0 International license (CC-BY 4.0)*
