# Supplementary material for: Utilisation of ChatGPT and other Artificial Intelligence tools among medical faculty in Uganda: a cross-sectional study
Source: MedEdPublish (2016). 2025 Apr 28;14:245. Originally published 2024 Oct 23. [Version 3] doi: 10.12688/mep.20554.3 (PMC11795020; doi:10.12688/mep.20554.3)
Supplement: Peer review history

# Reviewer report: Reviewer response for version 2

**DOI:** 10.21956/mep.22377.r40983

**Published:** 2025-02-04

**Authors:** Vijay Rajput, Jorge Cervantes

**Affiliations:** 1 Medical Education, Nova Southeastern University , Dr Kiran C. Patel College of Allopathic Medicine, Fort Lauderdale, FL, USA

The rapid growth of AI and GenAI has changed how country and continent are using the GenAI in their higher education. This one county study provide some basic understanding of usage and perception from Uganda. AI has existed before generative AI platforms such as GPTs, so authors should be careful in using the term “AI tools” when referring to GPTs.

 Data is from 2023, and during 2024 much has occurred in this field across the world. Learners and faculty are acquainted with GPTs, specially in universities and medical schools. Even certain GPTs names listed in the Introduction do not exist or modified by commercial entities . So in terms of bibliography, this manuscript need to update few important review papers and original studies which have addressed this very same question in African continent.(1)  This is a crucial aspect to be able to contrast their findings in the Discussion beyond Ref#3. For example, older faculty being less likely to use AI tools is something that has not been observed in the United States .  The Discussion is comparing their findings with studies done on doctoral studies and healthcare workers can have different outcome  and not easy to convince the reader.

 As many variables are compared, the inclusion of Table 2 in the Results is worth noticing. The authors can elaborate on the finding that the smaller medical schools (Busitema and Gulu) which have fewer students and faculty had not seen difference in ChatGPT usage. They only mention Makerere to have less usage in the Results when Table 2 is showing the same situation in Mbarara. The difference may be related to local sociocultural or access to GPT may require some more reflection in the discussion section. The responsible AI in Africa was published in 2022 getting into the challenges and opportunities of AI in African continent (2)  

 This study may need more updated information and new data from other part of Africa to see if there is any significant changes that has occurred in the last few years since the study was conducted.

  

1. Owoyemi A, Owoyemi J, Osiyemi A and Boyd A (2020) Artificial Intelligence for Healthcare in Africa.Front. Digit. Health 2:6. doi: 10.3389/fdgth.2020.00006
2. Okolo, Chinasa & Aruleba, Kehinde & Obaido, George. (2023). Responsible AI in Africa—Challenges and Opportunities. 10.1007/978-3-031-08215-3_3.

Have any limitations of the research been acknowledged?

Partly

Is the work clearly and accurately presented and does it cite the current literature?

Partly

If applicable, is the statistical analysis and its interpretation appropriate?

Yes

Are all the source data underlying the results available to ensure full reproducibility?

Partly

Is the study design appropriate and is the work technically sound?

Yes

Are the conclusions drawn adequately supported by the results?

Yes

Are sufficient details of methods and analysis provided to allow replication by others?

Partly

Reviewer Expertise:

Medical Education, Ethics, Artificial Intelligence

We confirm that we have read this submission and believe that we have an appropriate level of expertise to confirm that it is of an acceptable scientific standard, however we have significant reservations, as outlined above.

## References

[1] 1. Owoyemi A, Owoyemi J, Osiyemi A and Boyd A (2020) Artificial Intelligence for Healthcare in Africa.Front. Digit. Health 2:6. doi: 10.3389/fdgth.2020.00006.10.3389/fdgth.2020.00006PMC852185034713019

[2] 2. Okolo, Chinasa & Aruleba, Kehinde & Obaido, George. (2023). Responsible AI in Africa—Challenges and Opportunities. 10.1007/978-3-031-08215-3_3.

# Response

**Published:** 2025-04-19

**Authors:** Frank Kayemba

**Affiliations:** Faculty of Health Sciences, Busitema University, Tororo, Eastern Region, Uganda

I appreciate your expertise on this topic. I have read and understood your suggestions and made modifications accordingly.  

1. The study generally looks at generative AI. We use ChatGPT in the text to provide direction to the reader.
2. Many third-party applications are using GenAI, and this article focuses on the end-user applications, not the language model they use. For example, most of the applications listed use GPTs. (the list was generated from a qualitative study among medical faculty—unpublished data.)
3. The discussion section has been updated to cater to new findings in more recent studies.
4. Though there are differences in AI use among the universities, GenAI is accessed independent of the university; disparities in access are not expected. No university has publicly prohibited or discouraged the use of AI. There are no known sociocultural limitations to justify this finding.

# Reviewer report: Reviewer response for version 2

**DOI:** 10.21956/mep.22377.r40968

**Published:** 2025-01-27

**Authors:** Yavuz Selim Kıyak

**Affiliations:** 1 Department of Medical Education and Informatics, Gazi University, Ankara, Ankara, Turkey

The authors' amendments led to significant improvements in the paper. The new version of the manuscript barely reached a level that can be approved.

Have any limitations of the research been acknowledged?

Partly

Is the work clearly and accurately presented and does it cite the current literature?

Partly

If applicable, is the statistical analysis and its interpretation appropriate?

Yes

Are all the source data underlying the results available to ensure full reproducibility?

Yes

Is the study design appropriate and is the work technically sound?

Yes

Are the conclusions drawn adequately supported by the results?

Yes

Are sufficient details of methods and analysis provided to allow replication by others?

No

Reviewer Expertise:

Medical education, automatic item (question) generation using AI

I confirm that I have read this submission and believe that I have an appropriate level of expertise to confirm that it is of an acceptable scientific standard.

# Reviewer report: Reviewer response for version 1

**DOI:** 10.21956/mep.21999.r39952

**Published:** 2024-12-08

**Authors:** Li-Chun Chang

**Affiliations:** 1 Chang Gung University of Science and Technology, Kueishan, Taoyuan, Taiwan

This study adopts a straightforward cross-sectional design, with a few points that need further clarification:

 Study Setting

 Four public universities were selected, but the author should provide details on the number of faculty members, departments, and student faculty ratios at these universities. The use of ChatGPT may vary across different universities, yet this aspect has not been discussed by the author.

 Study Population

 The author needs to clarify whether the study population is "university staff" or "medical faculty." Are these two groups the same in this Information Classification: General study? How were the questionnaires distributed, and how much time was allowed for completion? What data were collected on work experience? Is it related to teaching experience? Some variables, such as department, were not included in the regression analysis. The author should explain the reasons for this omission.

Is the work clearly and accurately presented and does it cite the current literature?

Partly

If applicable, is the statistical analysis and its interpretation appropriate?

Yes

Are all the source data underlying the results available to ensure full reproducibility?

Partly

Is the study design appropriate and is the work technically sound?

Partly

Are the conclusions drawn adequately supported by the results?

Partly

Are sufficient details of methods and analysis provided to allow replication by others?

Yes

Reviewer Expertise:

Nursing education, use of ChatGPT

I confirm that I have read this submission and believe that I have an appropriate level of expertise to confirm that it is of an acceptable scientific standard, however I have significant reservations, as outlined above.

# Response

**Published:** 2025-01-20

**Authors:** Frank Kayemba

**Affiliations:** Faculty of Health Sciences, Busitema University, Tororo, Eastern Region, Uganda

Study setting

- The number of faculty, departments, and students per university has been included in the revised version. Notably, the description of "diploma" has been removed from the group of students because, much as it is a proper description of the university, the diploma school is independent. We only recruited faculty who teach undergraduate and postgraduate students.
- There were differences in the use of AI tools among the universities as listed in Table 2. However, we don't have reasons to justify these differences.

  Study population.

- The study population was medical faculty. Other university staff other than medical faculty were not included.
- No data were collected about the teaching experience.
- The questionnaires were distributed physically and filled out within three weeks. 
- The variable "departments" was not included in the regression because of the uneven distribution of faculty among the departments.

Thank you.

# Reviewer report: Reviewer response for version 1

**DOI:** 10.21956/mep.21999.r39957

**Published:** 2024-11-19

**Authors:** Yavuz Selim Kıyak

**Affiliations:** 1 Department of Medical Education and Informatics, Gazi University, Ankara, Ankara, Turkey

This is a cross-sectional study on the use of ChatGPT and some other AI tools among faculty members in four medical schools in a Global South country.

**Introduction:**

- “ChatGPT is an open-source large language model …” ChatGPT is a proprietary model.
- The authors should cite this BEME Review as it provides a comprehensive look on the topic: Gordon, M., Daniel, M., Ajiboye, A., Uraiby, H., Xu, N. Y., Bartlett, R., ... & Thammasitboon, S. (2024). A scoping review of artificial intelligence in medical education: BEME Guide No. 84. *Medical Teacher*, *46*(4), 446-470.
- The first two sentences in the last paragraph implies that the authors will not only report data on the use of AI tools but also report on using responsibly and will fill the gap the need for guidelines. However, the authors report only the use of AI tools. Therefore, the irrelevant content needs to be removed. The authors should only mention the gap that they try to fill: Data about the use of AI tools.
- This excellent brief source could guide the authors to revise the introduction section: Lingard, L. (2015). Joining a conversation: the problem/gap/hook heuristic. Perspectives on Medical education, 4, 252-253.

**Methods:**

- What was the total number of medical faculty members in each university?
- The authors should describe the development process of the data collection tool.
- Other models than ChatGPT and Gemini are not directly based on their AI models, they mostly are very specialized tools based on the existing AI models. For example, I could not understand why Snapchat AI is among the list considering the focus is medical education. The rationale behind presenting them in the survey rather than prominent AI models like Claude, Llama, Command R+, etc. should be provided. The omission of these prominent models should be added as a limitation.
- There is a very little information about the development process of the data collection tool. Please describe it in detail.
- The methods section should provide sufficient information that allows other researchers to replicate this study. An appropriate checklist among the reporting guidelines would be helpful for revising the section: https://www.equator-network.org/reporting-guidelines/

**Results:**

- "We interviewed ..." surveyed would be a better description as they filled a survey form.
- “28 used A tools for proofreading work” typo
- In figures, it is not clear if it shows only the frequencies or it shows percentages as well. Please make it clear.
- “Factors associated …” The authors reported age and university factors. Were there other significant factors? No matter there are or not, please state.

**Discussion:**

- Implications of the findings to medical education can be discussed in a brief paragraph.
- Limitations of the study should be expanded (e.g. the limitations in validity and comprehensiveness of the data collection tool, limited selection of AI tools, etc.).

Have any limitations of the research been acknowledged?

Partly

Is the work clearly and accurately presented and does it cite the current literature?

Partly

If applicable, is the statistical analysis and its interpretation appropriate?

Yes

Are all the source data underlying the results available to ensure full reproducibility?

Yes

Is the study design appropriate and is the work technically sound?

Yes

Are the conclusions drawn adequately supported by the results?

Yes

Are sufficient details of methods and analysis provided to allow replication by others?

No

Reviewer Expertise:

Medical education, automatic item (question) generation using AI

I confirm that I have read this submission and believe that I have an appropriate level of expertise to confirm that it is of an acceptable scientific standard, however I have significant reservations, as outlined above.

## References

[1] : A scoping review of artificial intelligence in medical education: BEME Guide No. 84. *Med Teach* .2024;46(4) : 10.1080/0142159X.2024.2314198 446-470 10.1080/0142159X.2024.2314198 38423127

[2] : Joining a conversation: the problem/gap/hook heuristic. *Perspect Med Educ* .2015;4(5) : 10.1007/s40037-015-0211-y 252-253 10.1007/s40037-015-0211-y 26346497 PMC4602011

[3] *Enhancing the QUAlity and Transparency Of health Research* . Reference source

# Response

**Published:** 2025-01-20

**Authors:** Frank Kayemba

**Affiliations:** Faculty of Health Sciences, Busitema University, Tororo, Eastern Region, Uganda

Thank you for the resources you shared and the comments. We have revised the article to include the recommended information.   Introduction

- ChatGPT is not open source; that has been corrected.
- The article only answers the gap of AI tools used by medical faculty, not policy. Writing about policy is only a justification of the need to know the former. I have paraphrased in that section to make this clear.

Methods

- The information about the number of faculty, students, and departments has been included in the revised version.
- Details on questionnaire development have been included as well. The list of AI tools listed in the questionnaire was based on a qualitative study—unpublished—exploring the AI tools used by medical faculty and students in Uganda; all AI tools mentioned in that study were included in the questionnaire. This also explains why other prominent models were not included. However, the question was multiple choice, and participants had the option to add more AI tools other than those listed. Even then, these models were not reported. I have added this to the limitations.

Results

- The use of the term "interviewed" was not proper; participants filled out a survey. This has been corrected.
- The figures have both frequencies (on the x-axis) and percentages (at each bar.)
- The factors for which regression was done are reported in Table 2. Other independent variables were left out on grounds of collinearity.

Discussion

- As reported above, the questionnaire was not exhaustive of all the major AI tools and hence limited content validity, I have included this among the limitations.
